# The 6-Minute Walk Test and Person-Reported Outcomes in Boys with Duchenne Muscular Dystrophy and Typically Developing Controls: Longitudinal Comparisons and Clinically-Meaningful Changes Over One Year

**DOI:** 10.1371/currents.md.9e17658b007eb79fcd6f723089f79e06

**Published:** 2013-07-08

**Authors:** Erik Henricson, Richard Abresch, Jay J. Han, Alina Nicorici, Erica Goude Keller, Evan de Bie, Craig M. McDonald

**Affiliations:** Department of Physical Medicine and Rehabilitation, University of California, Davis, Sacramento, California, USA; Department of Physical Medicine and Rehabilitation, University of California, Davis, Sacramento, California, USA; Department of Physical Medicine and Rehabilitation, University of California, Davis, Sacramento, California, USA; Department of Physical Medicine and Rehabilitation, University of California, Davis, Sacramento, California, USA; Department of Physical Medicine and Rehabilitation, University of California, Davis, Sacramento, California, USA; Department of Physical Medicine and Rehabilitation, University of California, Davis, Sacramento, California, USA; Department of Physical Medicine and Rehabilitation, University of California, Davis, Sacramento, California, USA

## Abstract

Introduction: Data is currently lacking anchoring a 30-meter longitudinal change in walking ability by 6-minute walk test (6MWT) in Duchenne muscular dystrophy as a minimal clinically important difference and “clinically meaningful” person-reported outcomes (PROs) at differing levels of ambulatory ability. Methods: We describe correlation between measures, 1-year change in measures, and correlation of 1-year changes between measures for the six-minute walk test (6MWT), 10-meter run/walk velocity, PedsQL and POSNA Pediatric Outcomes Data Collection Instrument (PODCI) in 24 4-12 year old. ambulatory DMD and 36 typical controls, and determine if minimal clinically important differences (MCID) of PROs contribute to different estimates of 6-minute walk distance (6MWD) change at differing levels of ability. Results: PedsQL total and physical function and PODCI global, transfer/mobility and sports/physical function PROs demonstrated significant differences between DMD and controls (p<0.00001). In DMD, 6MWD and 10-meter run/walk velocity were correlated with PODCI domain scores, with the transfer/mobility scale showing the strongest relationship (r=0.79 and r=0.76). In DMD, 6MWD distance and 10-meter run/walk velocity weakly correlated with PedsQL domain scores. In DMD, 6MWD, 10-meter run/walk velocity, and PODCI global and transfer and basic mobility demonstrated significant one-year change and exceeded the amount of change representing MCID. In DMD, 6MWD change highly correlated with change in PODCI global and PODCI transfer/mobility scores (r=0.76 and r=0.93). PODCI global and PODCI transfer/mobility scales provided the best estimates of 6MWT performance. A “meaningful” 4.5 point change in a low PODCI transfer / basic mobility score of 30 to 34.5 was associated with a 5.6m 6MWD change from 150.3 to 155.9m. At PODCI levels closer to normative levels for healthy controls, the change in 6MWD distance associated with a “meaningful” change in PODCI scores was almost 46m. Discussion: At lower levels of function, smaller increases in 6MWD result in meaningful change in quality of life (QoL) instrument scores. At higher levels of function, larger increases may be necessary to achieve the same QoL change score.

## Background

Duchenne muscular dystrophy (DMD) is an X-linked developmental disorder caused by mutations of the dystrophin gene^1^ and subsequent alteration or loss of production of dystrophin protein isoforms in muscle, central nervous system (CNS), and other tissues. Affecting approximately 1/3500 males^2^ , the disease is characterized by progressive loss of muscle strength that is frequently identified in the early toddler years as children begin to miss developmental motor milestones^3^ . Loss of dystrophin in CNS tissue is also evident in subpopulations of boys with DMD, although cognitive and sensorimotor sequelae of that loss are less well understood^4^ .

Prior to widespread use of glucocorticoids (GCs) as a primary method for maintaining muscle strength, mean age to loss of ambulation in DMD patients was around 9 years with an overall life expectancy into the mid-teen years or early twenties^5^ . Natural history studies of the disease published in the 1980s and early 1990s did not differ significantly from case reports by Duchenne a century earlier^6^ . Recent adoption of practice guidelines for GC use in ambulatory boys and a growing consensus that GCs may benefit nonambulatory boys as well through support of upper limb, cardiac, and respiratory function has contributed to an altered natural history of the disease, with decreased rates of progression, reductions in incidence of secondary conditions, and improved survival well into early adulthood^7^ .

While current practices have reduced rates of disease progression and have improved preservation of function for many with DMD, none of the available treatments has been directed toward restoration of the damaged gene or protein product. However, promising new therapies including nonsense mutation suppression via stop codon read through and exon skipping via antisense oligonucleotides and morpholinos have been introduced into registration-directed clinical trials using international multicenter collaborations. Both safety and efficacy trials have involved both ambulatory and nonambulatory subgroups of DMD populations^8^^,^ 9 ^,^ 10 . The research community has faced the need to identify and develop clinically meaningful outcome measures in DMD for use in pivotal therapeutic trials.

In boys with DMD, walking abnormalities are a major disease manifestation that has great importance to patients and families. Ambulation has been noted to be a prerequisite for normal physical functioning in humans^11^ ; the major goal of medical and physical therapy intervention during the ambulatory phase of DMD is to maintain ambulation for as long as possible^3 ^^,^ 12 ^,^ 13 ^,^ 14 . Given that ambulatory compromise is a key component of the DMD disease process and that ambulation measures the function of multiple muscle groups as well as cardiovascular activity, ambulation-related outcome measures are the most relevant endpoints in DMD patients who are still able to walk. We previously reported that the modified 6-minute Walk Test (6MWT) is feasible, safe, and reliable in boys with DMD who have not yet transitioned to full time wheelchair use^15^^,^ 16 . We also documented that they have markedly compromised ambulation relative to healthy boys and correlated 6-minute walk distance (6MWD) with age, anthropometric characteristics, and measures which change with disease progression, including stride length and cadence. In addition, 6MWD can be considered a proxy measure for the energy cost of locomotion in DMD^17^ . The 6MWT was been shown to be an integrated global measure of ambulatory function in DMD that is influenced by decreased lower extremity strength, biomechanical inefficiencies during gait, diminished endurance, and compromised cardio-respiratory status^17^ . Longitudinal data concerning the 6MWT in DMD have supported the clinically meaningful change in 6MWD to be in the range of 20 to 30 meters, which can serve as the targeted treatment effect in 12-month trials in ambulatory DMD^18^ . It appears that a decline of approximately 30 meters from an average performance on the 6MWT in DMD to a threshold 6MWD of < 325 meters or < 55%-predicted would place a patient with DMD at risk for more precipitous decline in ambulatory function over the subsequent year. Given the limitations of other measures in DMD including surrogate biomarkers, strength by myometry, and timed function tests (TFTs), the 6MWD has become the recommended primary outcome measure in ambulatory DMD.

Consumers, clinical researchers, the Food and Drug Administration (FDA), and industry have increasingly recognized the importance of person-reported outcome (PRO) measures in the determination of clinically meaningful outcomes and validation of clinical and surrogate endpoints for therapeutic trials^19^ . There are regulatory requirements that registration studies must incorporate primary endpoints that objectively measure clinically meaningful “life-changing” events with significant impact on health and well-being. In addition, the FDA has strongly recommended inclusion of PRO measures such as health-related quality of life assessments as an endpoints in all clinical trials^20^^,^ 21 .

The specific strength or function measures chosen to serve as the best primary and secondary clinical endpoints for a trial depends on the mechanism of action of the proposed therapeutic agent as well as the age and stage of disease targeted by the clinical trial design. Recently, investigators have cross-validated the 6MWD with many measures of strength and function historically used in DMD trials^17^, and also with other newer gross motor indices of disease progression in DMD such as the Northstar Ambulatory Assessment (NSAA)^22^ .

A potential limitation of the 6MWT and many other functional and strength-based performance measures is that while they do measure components of a clinically meaningful function (e.g., walking), they do not directly assess the patient’s health-related quality of life or participation in daily activities. While regulatory agencies have called for the inclusion of PRO measures in clinical trials, the sensitivity of these measures to short-term changes expected with treatment over a typical one year clinical trial duration has not been determined in DMD. The choice of PRO measures employed in DMD trials has unfortunately frequently been based on the availability of large normative data or translated test forms as opposed to natural history data that confirm that specific PRO measures are sensitive to expected treatment effects over the study duration. Expert groups in neuromuscular disease research and related fields have identified the need for development, validation and characterization of PRO measures of health, participation, and quality of life in different disease groups, as well as cross-validation of those measures with “traditional” clinical measures of strength and function^19^^,^ 23 ^,^ 24 .

In 2010, McDonald et al. 25 reported cross-sectional performance of traditional clinical outcome measures of lower limb function (Vignos functional scale, isometric knee extension strength, timed stand from supine, timed stair climb, walking velocity) in 56 ambulatory boys with DMD between 4 and 15 years of age against two commonly used health-related quality of life (HrQOL) measures, the Pediatric Quality of Life Inventory^TM^ (PedsQL)^26^^,^ 27 ^,^ 28 and the Pediatric Society of North America (POSNA) Pediatric Musculoskeletal Functional Health Questionnaire (also referred to as the Pediatric Outcomes Data Collection Instrument - PODCI)^29^^,^ 30 ^,^ 31 . We will refer to this instrument as the PODCI throughout this paper. The PedsQL is a 23-item patient self-report or parent proxy instrument designed to measure core health dimensions in children between 5 and 17 years of age. The instrument consists of four major scales, including physical functioning, emotional functioning, social functioning and school functioning. Items are scored in aggregate, for major scales independently, and as a psychosocial score including all but the physical function scale. The PODCI is a patient self-report or parent-proxy measure of functional ability in children with orthopedic limitations. Primary scales of the measure include upper extremity function, transfers and mobility, sports and physical function, comfort and pain, happiness and satisfaction, and expectations for treatment. In their study, McDonald et al demonstrated that both measures differentiate between boys with DMD and typically-developing controls, and that there are age-related effects associated with DMD disease severity on PedsQL physical function and PODCI transfer and basic mobility, sports and physical function and global scales^25^ . They also demonstrated moderate correlation of those PODCI scales (r=0.38-0.53) and weaker correlation of the PedsQL physical function scale (r=0.2-0.41) to variations in disease involvement as measured by functional evaluations, quantitative strength and functional evaluation. Those correlations demonstrated a relationship between patient-reported HrQOL and clinician-measured functional performance tools, and lent support to the concept that those commonly used tools are “clinically meaningful” in the context of a patient’s day-to-day life. The authors cited a need for additional longitudinal data to assess responsiveness of those measures over time in order to determine the utility of the HrQOL tools as outcome measures in clinical trials.

Given the demonstration of a moderately strong relationship between HrQOL and physical function, it stands to reason that it may not always be accurate to assume that a uniform 30-meter change in walking ability by 6MWT will always be clinically meaningful from the standpoint of an individual’s self-reported abilities and QoL. Instead, it may be the case that smaller or larger changes in ability will contribute to meaningful patient-reported function at differing levels of baseline ability.

## Specific Aims

In the current study, we describe the longitudinal characteristics of the 6MWT, PedsQL, and PODCI instruments in ambulatory boys with DMD and typically-developing controls. We set out to: 1) further characterize correlation between measures at baseline for ambulatory DMD boys and controls, as well as the combined ranges of both; 2) describe significant 1-year change in the measures, and to describe the correlation of 1-year changes between the measures; and 3) determine whether regression estimates could be used to describe relationships between measures over time. Fourth, we further aimed to describe the estimated minimal clinically important difference (MCID) for each measure using baseline statistical distribution properties, and to determine whether the MCIDs of HrQOL measures would be associated with different estimates of meaningful changes in walking distance across the spectrum of walking abilities in DMD.

## Participants and Methods

***Study Subjects***

The institutional review board (IRB) reviewed and approved the study protocol. Informed consent/assent was obtained for each participant prior to initiation of any study procedures. Participants included boys with DMD and typically developing controls between the ages of 4 and 12 years. Boys with DMD were required to have a clinical picture consistent with the disease and confirmation by at least one of the following methods: 1) documentation of a mutation of the dystrophin gene consistent with a diagnosis of DMD; 2) complete loss of dystrophin on muscle biopsy; or 3) elevated serum creatine kinase and a family history of DMD in a primary male relative who satisfied conditions (1) or (2). All participants were required to be able to walk ≥10 meters, be free of any medications that would affect heart rate or metabolism, and be free of concomitant illness or contraindications to exercise. All participants had to be able to follow simple instructions. English- and Spanish-speaking boys with DMD were recruited from our neuromuscular disease clinic and the northern California region. Typically-developing controls were recruited through locally-posted English and Spanish advertisements.

***Evaluation Methods***

Methods for the DMD-specific Six-minute Walk Test (6MWT) have been previously reported by our group^15^^,^ 16. The 6MWT was administered at baseline at the end of a test battery that also included anthropometric measures, timed standing from supine, timed 4-stair climb, and timed 10- and 25-meter walk/run tests as previously described. Participants viewed a short orientation video that was developed to address potential attention and verbal working memory limitations, and then underwent testing on a 25-meter indoor course. Participants were instructed to travel as far and as fast as possible in six minutes without running in a manner that demonstrated consistency with published normative studies^32^^,^ 33. Participants were provided with regular periodic verbal encouragement, and were followed by a “safety” chaser who responded in the event of a fall. PedsQL and PODCI instruments were provided as paper-based reports in English or Spanish and were completed by the parent or primary caregiver during the evaluation visit. Instrument scores were compiled according to scoring paradigms published with each instrument. Participants were evaluated at baseline and using identical methods at a 1-year follow-up visit.

***Statistical Methods***

Because this was a pilot study with no *a priori* hypotheses, we sought to enroll at least 20 participants in the DMD and control arms of the study, with a balance of children in 4-6, 7-9 and 10-12 year age groups at enrollment. All data was transformed as necessary to meet assumptions of OLS regression, and were back-transformed for reporting purposes. P-values <0.05 were considered statistically significant. Baseline characteristics were summarized using standard descriptive statistics, and differences between groups were assessed using standard between-group mean comparison tests. MCID was calculated at 1/3 of a measure’s standard deviation for the DMD group at the baseline evaluation^34^ . Correlation of measures at baseline were calculated using the Pearson method. Annual change scores for each measure were calculated as the difference between the baseline and 1-year visits; significance of change scores was evaluated using one-way analysis of variance with visits 1/2 and DMD/control as grouping variables. Correlation of change between measures was calculated using the Pearson method. To evaluate the relationship between 6MWD and HrQOL measures, we sequentially evaluated multiple regression models from our data using PedsQL and PODCI domains previously identified by McDonald et al.^25^ to demonstrate a relationship between physical function and participation as well as age at evaluation, height, and weight that have been demonstrated to affect predicted values of 6MWD^32^^,^ 33 . In selecting our model, we determined our “best fit” equation based on: 1) presence of only statistically significant (p<0.05) explanatory variables in the model; 2) maximized r^2^ statistic, and 3) the highest significant F statistic with the fewest degrees of freedom in the model. To evaluate differences in 6MWD MCID using the “best fit” model, we calculated predicted 6MWD values at the mean of age, height, and weight that represented upper and lower limits of the MCID range of the HrQOL instrument across the observed range of 6MWT performance (range of 6MWD).

## Results

Twenty-four boys with DMD and 36 typically-developing control boys between the ages of 4 and 12 years of age underwent evaluation at baseline. Descriptive statistics and MCID values for outcome measures in DMD and control participants is shown in Table 1. The 10 meter run/walk velocity was also assessed because of the high correlation between this readily obtainable secondary endpoint for ambulatory function and the 6MWD^17^^,^ 18 . The groups did not differ significantly by age or weight, although DMD boys were on average 10cm shorter than controls, which is consistent with previous reports. Also consistent with previous reports^25^ , the 6MWD, 10 meter run/walk velocity, PedsQL total and physical function scores and the PODCI global scor, transfer and basic mobility score, and sports and physical function score all demonstrated significant differences between DMD and control boys (p<0.00001).

**Table 1: Descriptive statistics at baseline:** 

	DMD			Control		
Variable	Mean	SD	MCID	Mean	SD	*P*
Age (years)	7.9	2.3		8.7	2.6	*ns*
Height (cm)	123.0	14.8		133.4	16.6	0.0087
Weight (kg)	33.2	16.6		35.5	17.2	*ns*
6MWD (meters)	369.5	79.3	26.4	613.3	73.6	0.0001
10-meter run/walk velocity (m/s)	1.68	0.56	0.19	3.32	0.36	0.0001
PedsQL Total Score	57.3	10.1	3.5	88.8	10.6	0.0001
PedsQL Physical Function Score	48.0	20.0	6.7	92.9	12.5	0.0001
PODCI Global Score	73.8	11.5	3.8	97.9	3.0	0.0001
PODCI Transfer / Basic Mobility Score	79.5	13.5	4.5	100.3	1.6	0.0001
PODCI Sports and Physical Function Score	55.0	21.2	7.1	95.8	5.8	0.0001

Correlation are shown between the 6MWD and PROs (Table 2) and the 10 meter run/walk velocity and PRO measures (Table 3). In boys with DMD, 6MWD was strongly correlated with PODCI measures, with the transfer and basic mobility scale showing the strongest relationship (r=0.79). In addition, the 10 meter run/walk velocity was also strongly correlated with the PODCI measures, including the transfer and basic mobility scale (r=0.76). In the DMD group 6MWD and 10 meter run/walk velocity was weakly correlated with PedsQL items. In controls, PedsQL physical function and PODCI global score showed weak correlations with ambulatory measures. Across the full range of both 6MWD and HrQOL measures with the groups combined, 6MWD demonstrated strong to very strong correlations with both PODCI and PedsQL measures (r=0.85-0.88 and r=0.71, respectively). Similarly, across the full range of both 10 meter run/walk velocities and PROs, the 10 meter run/walk velocity demonstrated very strong correlations with both PODCI and PedsQL measures (r=0.78-0.82 and r=0.71-0.72, respectively).

**Table 2: Pearson's Correlation of 6MWD and PedsQL and PODCI Measures:** 

6MWD vs. Person-Reported Measures	DMD	Control	Combined (Full Range)
PedsQL Total Score	0.21	-0.02	0.71
PedsQL Physical Function Score	0.18	0.19	0.71
PODCI Global Score	0.68	0.28	0.88
PODCI Transfer / Basic Mobility Score	0.79	0.01	0.86
PODCI Sports and Physical Function Score	0.68	0.12	0.85

**Table 3: Pearson's correlation of 10-meter run/walk velocity and PedsQL and PODCI measures:** 

10-meter run/walk velocity vs. person-reported measures	DMD	Control	Combined (Full Range)
PedsQL Total Score	0.15	0.14	0.71
PedsQL Physical Function Score	0.21	0.23	0.72
PODCI Global Score	0.62	0.28	0.82
PODCI Transfer / Basic Mobility Score	0.76	-0.05	0.78
PODCI Sports and Physical Function Score	0.58	0.29	0.82

The distribution of 6MWD versus PRO measures are shown for PODCI transfer and basic mobility scale scores (Figure 1a), PedsQL physical function scores (Figure 1b), PODCI global scores (Figure 2a) and PedsQL total scores (Figure 2b). The distribution of 10 meter run/walk velocities versus PRO measures are shown for PODCI transfer and basic mobility scale scores (Figure 3a), PedsQL physical function scores (Figure 3b), PODCI global scores (Figure 4a) and PedsQL total scores (Figure 4b).


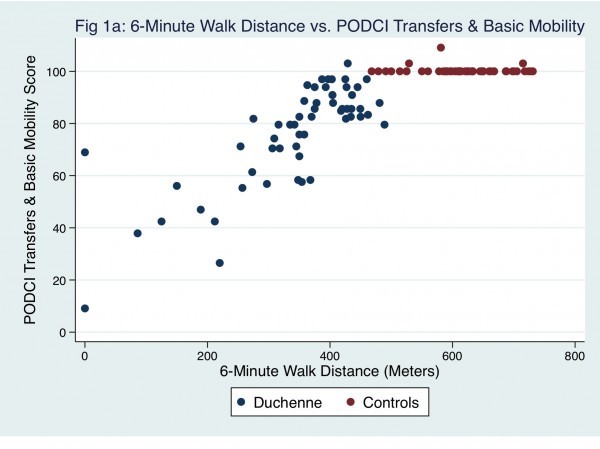



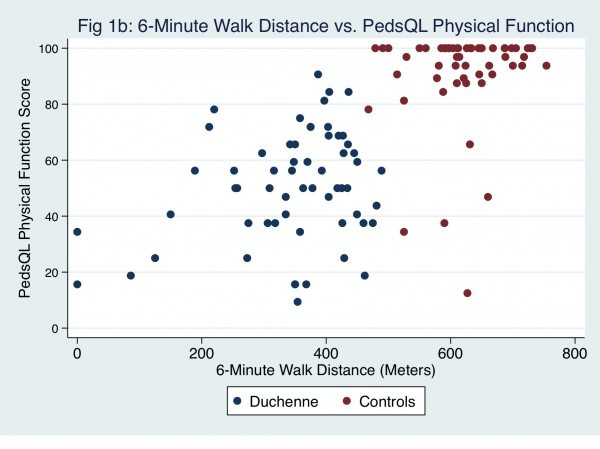



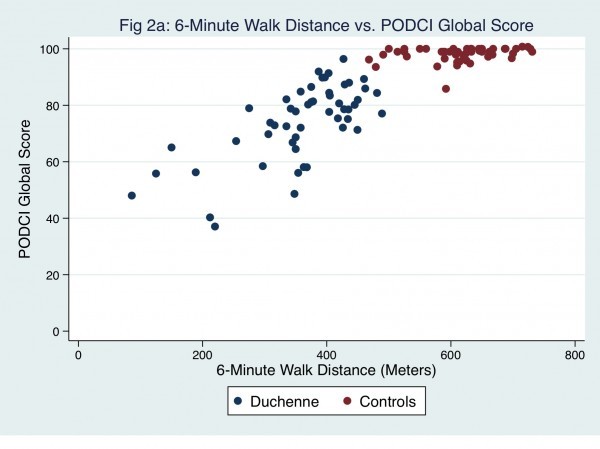



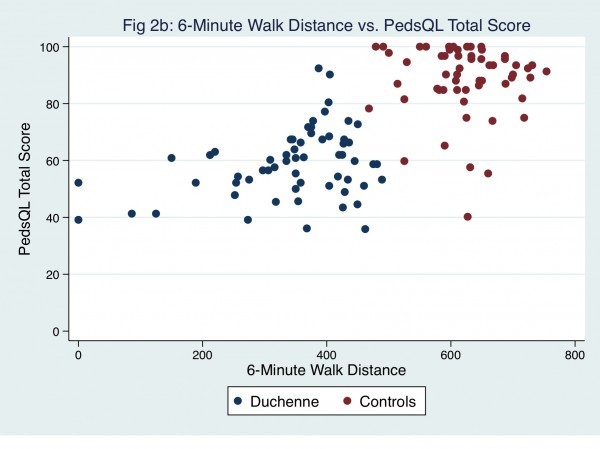



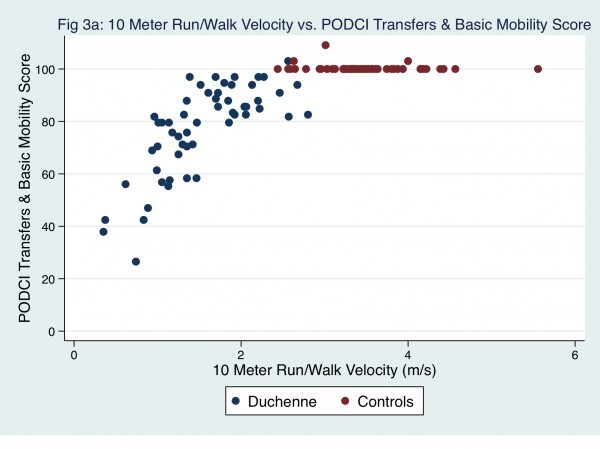



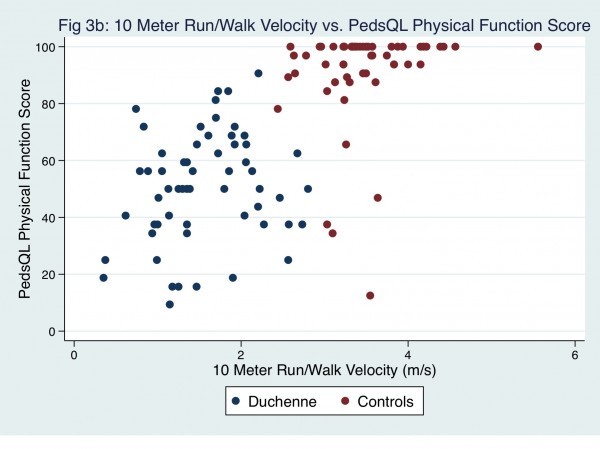



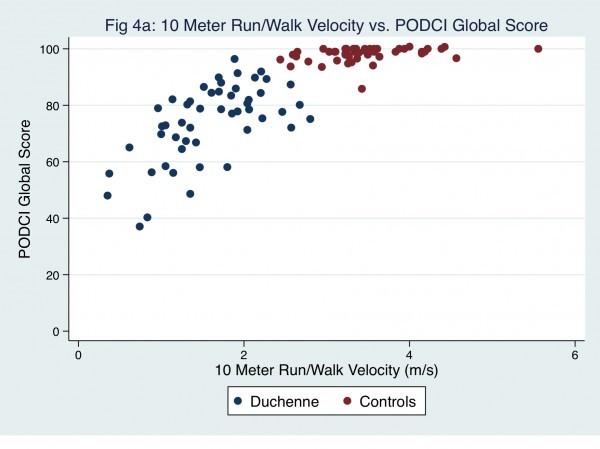



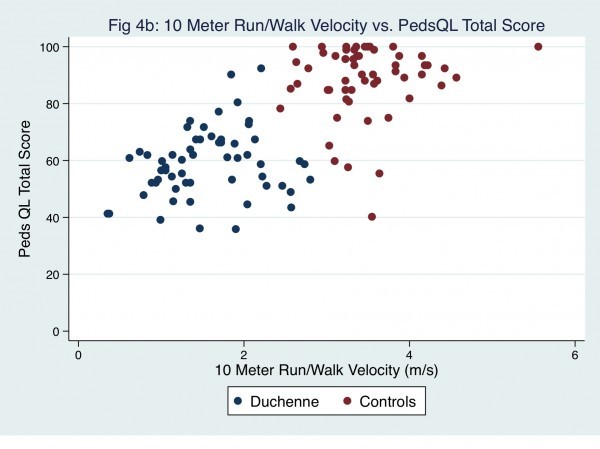


One-year follow-up scores were obtained in 13 DMD and 18 control participants. Annual mean change scores for 6MWD, 10 meter run/walk velocity, and HrQOL measures are shown in Table 4. In boys with DMD, only 6MWD, 10-meter run/walk velocity, and PODCI global and transfer and basic mobility scales demonstrated statistically significant change over the 1-year interval. In DMD subjects, the natural history change for those four tests exceeded the amount of change estimated to represent the MCID using baseline values. The control group demonstrated significant 1-year change in only 10 meter run/walk velocity.

**Table 4: 1-year change in 6MWD, 10-meter run/walk velocity and person-reported functional measures:** 

	DMD (n=13)				Control (n=18)		
**Outcome Measure**	**Mean**	**SE**	**p**	**> MCID**	**Mean**	**SE**	**p**
6MWD (meters)	-53.67	25.96	0.027	Yes	16.5	11.46	*ns*
10-meter run/walk velocity (m/sec)	-0.25	0.68	0.007	Yes	0.33	0.07	0.0001
PedsQL Total Score	-0.13	2.67	*ns*	No	1.03	3.26	*ns*
PedsQL Physical Function Score	-2.08	6.96	*ns*	No	3.74	4.71	*ns*
PODCI Global	-5.05	2.24	0.027	Yes	0.43	0.46	*ns*
PODCI Transfer / Basic Mobility	-9.95	3.94	0.013	Yes	0.31	0.51	*ns*
PODCI Sports	-3.11	4.04	*ns*	No	1.49	0.99	*ns*

Correlation of 1-year changes in 6MWD and HrQOL measures is shown in Table 5. In boys with DMD, change in 6MWD was most highly correlated with change in PODCI global and PODCI transfer and basic mobility scores (r=0.76 and r=0.93, respectively).

**Table 5: Pearson's correlation of 1-year change in 6MWD, PedsQL and PODCI measures:** 

6MWD vs. Person-Reported Measures (Change)	DMD (n=13)	Control (n=18)	Combined (Full Range, n=31)
PedsQL Total Score	0.01	-0.13	-0.17
PedsQL Physical Function Score	-0.36	-0.06	-0.38
PODCI Global Score	0.76	0.07	0.68
PODCI Transfer / Basic Mobility	0.93	-0.05	0.76
PODCI Sports	0.45	-0.60	0.37

Best-fit regression equations estimating 6MWD for each of the HrQOL items is listed in Table 6. Consistent with the higher degrees of correlation between 6MWD and PODCI measures and change in those measures over time, the PODCI global and PODCI transfer and basic mobility scales provided the best estimates of 6MWT performance. We selected the latter to best represent the relationship between 6MWD and HrQOL measures due to the PODCI global score being derived in part from (and thus somewhat collinear with) the transfer and basic mobility score.

**Table 6: Best-fit regression equations for mobility, strength and function:** 

Instrument	Equation	R-Squared	Prof>F
PedsQL Total	6MWD = 5.39(PedsQL Total Score)+4.4(Height cm)-2.41(Weight kg)-399.55	0.58	0.00001
PedsQL Physical Function	6MWD = 3.67(PedsQL Physical Function Score)+3.87(Height cm)-2.21(Weight kg)-199.99	0.56	0.00001
PODCI Sports	6MWD = 0.0003(PODCI Sports^3^)+3.35(Height cm)-1.90(Weight kg)-56.35	0.79	0.00001
PODCI Global	6MWD = 0.0004(PODCI Global^3^)+3.42(Height cm)-2.31(Weight kg)-158.36	0.83	0.00001
PODCI Transfer / Basic Mobility	6MWD = 0.0004(PODCI T&M^3^)+4.41(Height cm)-2.80(Weight kg)-310.79	0.83	0.00001

Using the baseline calculated MCID for the PODCI transfer and basic mobility score of 4.5 points and mean height and weight for the DMD group, we calculated 6MWD from a range of PODCI scores to demonstrate the degree of 6MWD change that would be associated with a “meaningful” change in parent-proxy-related PODCI scores (Table 7). From a HrQOL-based perspective, a “meaningful” change in mobility might be related to small changes in walking distance at lower levels of function. For instance, a 4.5 point change in PODCI transfer and basic mobility score from 30 to 34.5 is associated with a 5.6m 6MWD change from 150.3 to 155.9m. At the other end of the spectrum, PODCI levels closer to normative values and associated with higher 6MWD values would require a greater change in 6MWD over one-year to affect a “meaningful” change of 4.5 in PODCI transfer and basic scores. A 4.5 point change at this higher level of ambulatory function would be associated with a 6MWD change of almost 46m.

**Table 7: Change in 6MWD necessary at different ranges of function to achieve a 4.5 point (MCID) change in PODCI Transfer and Basic Mobility scale score:** 

PODCI Range (pts)	6MWD Range (m)	6MWD Change (m)
30 - 34.5	150.3 - 155.9	5.6
40 - 44.5	165.0 - 174.7	9.7
50 - 54.5	189.5 - 202.2	12.7
60 - 64.5	225.9 - 246.8	20.9
70 - 74.5	276.7 - 304.9	28.2
80 - 84.5	344.3 - 380.8	36.5
90 - 94.5	431.1 - 477.0	45.9

## Discussion

**The PODCI instrument is more sensitive to DMD disease progression than the PedsQL.**

Here we expand on previous work by McDonald and colleagues^25^ to demonstrate relationship between clinician-measured outcomes of function (6MWT and 10 meter run/walk velocity) and parent-reported HrQoL. In support of previous reports, both PedsQL and PODCI significantly differentiate between DMD and typically-developing control subjects. In addition, in a multicenter international clinical trial which included 174 DMD subjects over a wider range of ambulatory abilities, a modest correlation was found between the 6MWD and PedsQL physical scale^17^ . In the current study, we demonstrate that the PODCI instrument global, transfer and basic mobility, and sports physical function scales are more strongly correlated with 6WMD than PedsQL total and physical function scale scores. Similarly, one-year changes in PODCI scores are more strongly correlated with 1-year change in 6MWD than are PedsQL scores. These data provide support for the concept that functional performance measures of ambulation in DMD such as the 6MWT and 10 meter run/walk velocity are “clinically-meaningful.” These longitudinal data show that while PODCI and PedsQL both demonstrate associations with disease severity in DMD, the PODCI instrument’s increased sensitivity to change makes it better suited for use as an outcome measure in clinical trials due its ability to show significant change over a 1-year time period commonly employed in current clinical trial designs.

The key to this increased level of sensitivity to change associated with disease progression of the PODCI may lie in the design of the instruments themselves, which differ in their approach to self/proxy-reported ability. The PODCI items are framed in terms of ease or difficulty of a task, such as: “During the last week has it been easy or hard for your child to walk one block?” The response options address the perceived physical difficulty of activities: “easy", "a little hard", "very hard", or "can't do at all”. In contrast, the PedsQL framework addresses the frequency of difficulty with activities. Items are presented such as “In the past ONE month how much of a problem has your child had with walking more than one block?” Response choices include: “never", "almost never", "sometimes", "often", and "almost always.” The PedsQL represents a subtly different and more participation-oriented construct that accounts for not only perceived problems with an activity, but the self-selected frequency or importance as well. This subtle difference helps to explain why PODCI transfer and basic mobility items scale items correlate better with objective clinical measures of function such as 6MWD or 10 meter run/walk velocity and are more sensitive to changes in those abilities over time. They represent a purer measure of locomotor ability that is less affected by behavior.

**Changes in PODCI responses and walking velocity correlate strongly, but “clinical meaningfulness” may differ across ranges of performance.**

Regression estimates from this sample allow us to predict 6MWD based on PODCI score, and subsequently we can estimate differences between MCID for each measure at differing levels of function. Using our regression estimates, it is possible to conclude that a clinically-important difference in person-reported HrQOL via PODCI transfer and basic mobility score is associated with a decreasing degree of change in achievable 6MWT velocities or distances as baseline function decreases. Thus, the degree of perceived “meaningful change” in walking ability may differ according to functional capacity. Hence at higher levels of function on the 6MWT closer to healthy control values, it may take a greater change in 6MWD to produce clinically-meaningful changes on a PRO measure such as the PODCI transfer and basic mobility scale. At most levels of function typically seen in ambulatory DMD subjects, the threshold change in 6MWD which would correspond to clinically meaningful changes in the PODCI transfer and basic mobility scale would be up to 28 to 36 meters. For example, at a mean 6MWD for DMD of 356 meters documented in one trial^17^, the change in 6MWD associated with a clinically-meaningful 4.5 point change in PODCI transfer and basic mobility scale would be 36 meters. At a 300 meter 6MWD that seems to represent a lower limit threshold level of function placing DMD subjects at risk for loss of ambulation^18^ , the MCID of 4.5 points on the PODCI corresponds to a change in 6MWD of 28 meters. These are levels of ambulatory ability that are frequently seen in clinical trials for ambulatory DMD boys, providing support for the 6MWD of 30m MCID and effect size that has been commonly proposed as a basis for study power calculations. However, at low levels of function, smaller increases or changes due to treatment in achievable walking distance are likely to result in “meaningful” changes in HrQoL.

## Conclusions

There is ongoing development of regulatory agency guidance regarding the most appropriate measures for use as primary or co-primary endpoints for clinical trials for individuals affected by Duchenne and Becker muscular dystrophies^35^. A major facet of that discussion revolves around what endpoints, whether of muscle strength, functional capacity or QoL should be used as *a priori* measures of “meaningful” success in clinical trials. Results of this small preliminary study suggest that the PODCI instrument is superior to the PedsQL measure with regard to sensitivity to change over a 1-year period and is an appropriate PRO measure of both function and HrQoL in DMD; confirmation in a larger DMD cohort is currently underway. Our early results, however, suggest that in less functional individuals, small changes in household walking ability may represent a change in daily function that could have a significant perceived impact on the individual. Conversely, at higher levels of function closer to the norm, larger increases in achievable walking distance are necessary to gain a “meaningful” increase in HrQoL. At performance levels closer to normative abilities that might be seen in milder Becker dystrophinopathies, changes in 6MWD needed to affect a “meaningful” change in PODCI scores could be much larger and possibly outside of the level of change expected in a 1-year clinical trial. We therefore suggest that regardless of whether clinician-derived functional measures and HrQoL measures are used in concert or independently in clinical investigations, interpretation of achieved change in walking distances or ambulatory velocity in clinical trials in DMD may vary by age or the magnitude of difference compared to healthy controls, and that these factors may affect power calculations and sample sizes needed for clinical trials.

## Competing Interests

E.K. Henricson: Mr. Henricson has served as a member of the Cooperative International Neuromuscular Research Group (CINRG) Executive Committee, and as a consultant for Genzyme, Inc. and PTC Therapeutics, Inc.

R.T. Abresch: Mr. Abresch has served as a consultant for PTC Therapeutics, Inc.

J.J. Han: Dr Han has served as a consultant for Genzyme, Inc.

A. Nicorici: Ms. Nicorici has nothing to disclose.

E. Goude: Ms. Goude has nothing to disclose.

E. de Bie: Mr. de Bie has nothing to disclose.

CM McDonald: Dr. McDonald is a member of the Cooperative Neuromuscular Research Group (CINRG) Executive Committee and has served on advisory committees for PTC Therapeutics, Inc., Sarepta Therapeutics, Inc., GlaxoSmithKline, PLC., Prosensa, Halo Therapeutics, Shire HGT, Cardero Therapeutics, Novartis AG, and Eli Lilly and Company.

## Statistical Analysis

Erik K. Henricson, UC Davis Department of Physical Medicine & Rehabilitation

## Corresponding Author

Erik K. Henricson, MPH

University of California, Davis School of Medicine

Sacramento, California 95817

Telephone: 916-734-0384

Fax: 916-734-7838

E-mail: erik.henricson@ucdmc.ucdavis.edu

## Key Words / MESH Terms

Child (Preschool), Child, Adolescent, Disease Progression, Follow-Up Studies, Gait/physiology*, Humans, Male, Muscular Dystrophy, Duchenne/physiopathology*, Walking/physiology*, Quality of Life, Marker/clinical, Marker/surrogate. 6-minute walk test, 6MWT, PedsQL, PODCI
